# Intervention to Improve Appropriate Prescribing and Reduce Polypharmacy in Elderly Patients Admitted to an Internal Medicine Unit

**DOI:** 10.1371/journal.pone.0166359

**Published:** 2016-11-30

**Authors:** Milena Urfer, Luigia Elzi, Salome Dell-Kuster, Stefano Bassetti

**Affiliations:** 1 Division of Internal Medicine, Kantonsspital Olten, Olten, Switzerland; 2 Division of Infectious Diseases, Regional Hospital San Giovanni, Bellinzona, Switzerland; 3 Basel Institute for Clinical Epidemiology and Biostatistics, University Hospital Basel, Basel, Switzerland; 4 Division of Internal Medicine, University Hospital Basel, Basel, Switzerland; 5 Department of Clinical Research, University Hospital Basel, Basel, Switzerland; University of Sydney, AUSTRALIA

## Abstract

**Background:**

Polypharmacy and inappropriate medication prescriptions are associated with increased morbidity and mortality. Most interventions proposed to improve appropriate prescribing are time and resource intensive and therefore hardly applicable in daily clinical practice.

**Objective:**

To test the efficacy of an easy-to-use checklist aimed at supporting the therapeutic reasoning of physicians in order to reduce inappropriate prescribing and polypharmacy.

**Methods:**

We assessed the efficacy and safety of a 5-point checklist to be used by all physicians on the internal medicine wards of a Swiss hospital by comparing outcomes in 450 consecutive patients aged ≥65 years hospitalized after the introduction of the checklist, and in 450 consecutive patients ≥65 years hospitalized before the introduction of the checklist. The main measures were the proportion of patients with prescription of potentially inappropriate medications (PIMs) at discharge, according to STOPP criteria, and the number of prescribed medications at discharge, before and after the introduction of the checklist. Secondary outcomes were the prevalence of polypharmacy (≥ 5 drugs) and hyperpolypharmacy (≥ 10 drugs), and the prevalence of potentially inappropriate prescribing omissions (PPOs) according to START criteria.

**Results:**

At admission 59% of the 900 patients were taking > 5 drugs, 13% ≥ 10 drugs, 37% had ≥ 1 PIM and 25% ≥ 1 PPO. The introduction of the checklist was associated with a significant reduction by 22% of the risk of being prescribed ≥ 1 PIM at discharge (adjusted risk ratios [RR] 0.78; 95% CI: 0.68–0.94), but not with a reduction of at least 20% of the number of drugs prescribed at discharge, nor with a reduction of the risk of PPOs at discharge.

**Conclusions:**

The introduction of an easy-to-use 5-point checklist aimed at supporting therapeutic reasoning of physicians on internal medicine wards significantly reduced the risk of prescriptions of inappropriate medications at discharge.

## Introduction

Because of the high prevalence of comorbid diseases in the higher aged, many elderly people are treated with multiple medications. The proportion of older adults exposed to polypharmacy (usually defined as concomitant prescription of ≥ 5 drugs) is rapidly increasing in the last years. This increase has several reasons, such as the rising use of cardioprotective and antidepressant medications [1] and probably also the promotion of guidelines recommending multiple drug therapy to achieve targets such as blood pressure or glycaemic control [2]. Approximately 20% to 40% of adults aged 65 and older in developed countries are prescribed ≥ 5 medications [1, 2]. Whereas polypharmacy is driven by comorbidity and may be beneficial for many patients, the number of medications used is the strongest risk factor for prescribing problems [3]. Polypharmacy results in medication nonadherence, and increases the risk of adverse drug reactions, drug-drug interactions, medication errors and of using potentially inappropriate medications (PIMs) [1]. Several studies showed that polypharmacy and inappropriate medication use are associated with adverse health outcomes, including mortality, hospitalization, falls and cognitive impairment [4–7].

Many interventions aiming at assessing and reducing the number of inappropriate medications and at optimizing appropriate prescribing in elderly people have been proposed. Appropriateness of prescribing can be assessed by explicit (criterion-based) or implicit (judgment-based) outcome measures [8]. Explicit indicators, such as lists of drugs that should be avoided in elderly people (e.g. the Beers list [9]), are usually drug-oriented or disease-oriented, do not address the burden of comorbid diseases in the individual patient and are limited due to prescribing habits across countries [8, 10]. With implicit, judgment-based approaches, clinicians use information from the patient and published work (instead e.g. of a fixed list of to-avoid-drugs) to make judgements about appropriateness. These approaches address multiple elements of medication prescribing, which are relevant for many different drugs, clinical conditions and settings [11]. They are flexible, focus on the patient, rather than on drugs or diseases, and are potentially the most sensitive, but they are time-consuming and depend on the user’s knowledge and experience [8, 11]. Deprescribing, the systematic process of identifying and tapering or discontinuing drugs in patients in which potential harms outweigh potential benefits has also been proposed as tool to reduce inappropriate polypharmacy [12]. However, most interventions described to improve appropriate prescribing in elderly people are complex, based on explicit criteria and require the presence of clinical pharmacists and/or multidisciplinary teams including for example geriatricians and other healthcare providers with specialized geriatrics training (e.g. nurses, pharmacists, psychiatrists) [8, 13]. These resources are not available in many settings. We therefore developed an intervention aimed at systematically integrating the key judgment-based elements for appropriate prescribing in the ongoing process of clinical reasoning regarding each individual patient. We used and simplified two published conceptual frameworks aiming at minimizing inappropriate medications in elderly people [14, 15] and created a checklist to be used by physicians on internal medicine wards. Finally, we assessed the efficacy and safety of this intervention and showed that the introduction of the checklist reduced the risk of prescription of inappropriate medications at discharge.

The primary aim of the study was to assess the efficacy and safety of a prescriber checklist for reducing inappropriate prescribing and polypharmacy among patients aged ≥ 65 years admitted to an internal medicine unit. Secondary aims were to assess the number of prescribed drugs, the prevalence of polypharmacy (concomitant use of ≥ 5 drugs) and hyperpolypharmacy (concomitant use of ≥ 10 drugs), to assess the prevalence of potentially inappropriate medications (PIMs) and potentially inappropriate prescribing omissions (PPOs), and to assess the prevalence of prescription and the rate of inappropriate prescription of following drugs: non-steroidal anti-inflammatory drugs (NSAID), proton pump inhibitors (PPI), systemic corticosteroids, metamizole (dipyrone) and potent opiates.

## Methods

**Fig 1:**
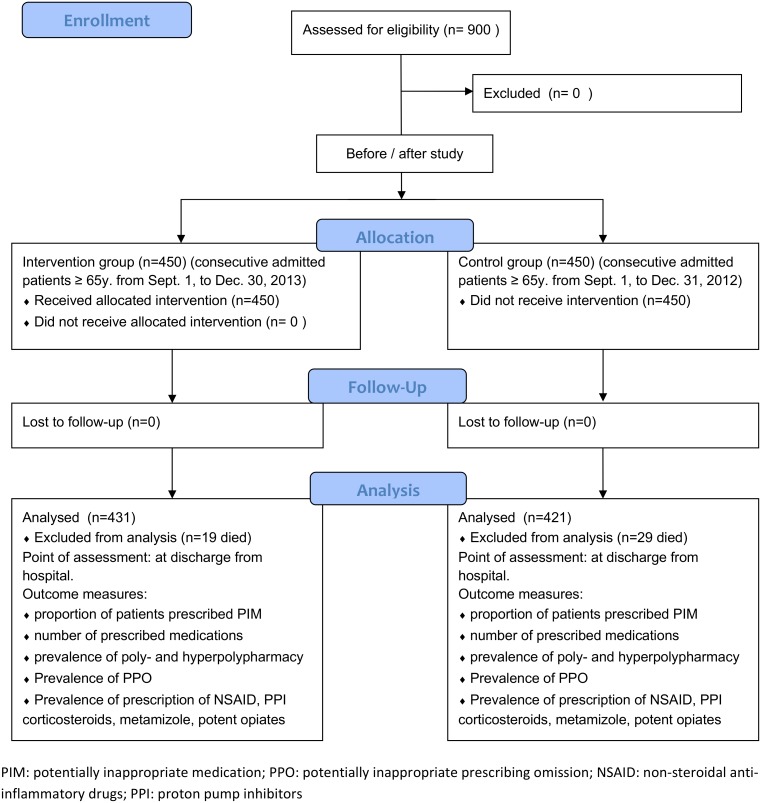
CONSORT Flow Diagram.

We conducted a single-center, interventional, quasi-experimental before-after study in the Division of Internal Medicine of the Kantonsspital Olten, a university-affiliated secondary-level teaching hospital with 245 beds in northwestern Switzerland. To detect an effect of 10% of the checklist (decrease in prevalence of inappropriate drug prescription and/or polypharmacy from anticipated 30% at admission to 20% at discharge) with a probability (power) of 90% at a significance level (alpha, 2-tailed) of 0.05, a sample size of 824 patients (412 in each group) is needed. Therefore, we included in the analysis the first 450 consecutive patients aged ≥ 65 years hospitalized in the Division of Internal Medicine during the period September 1^st^–December 30^th^, 2013, after the introduction of the checklist (intervention group), and compared them to the first 450 consecutive patients aged ≥ 65 years hospitalized in the same division during the same period of the previous year (September 1^st^–December 31^th^, 2012) (control group) (Fig 1). The consecutive patients were identified through the admission lists generated by the electronic hospital information system. Each patient was included only once (at the first hospitalization). Patients who died during the hospitalization were excluded from further analysis.

**Fig 2:**
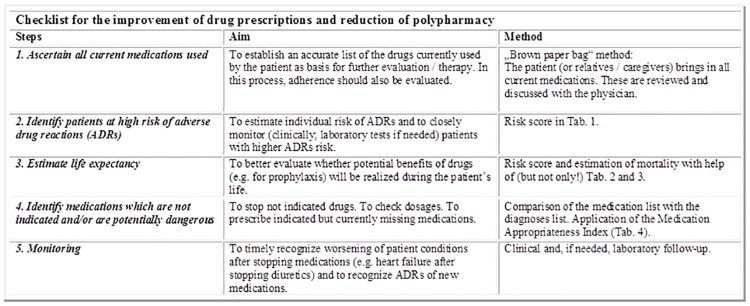
Checklist, page 1.

**Fig 3:**
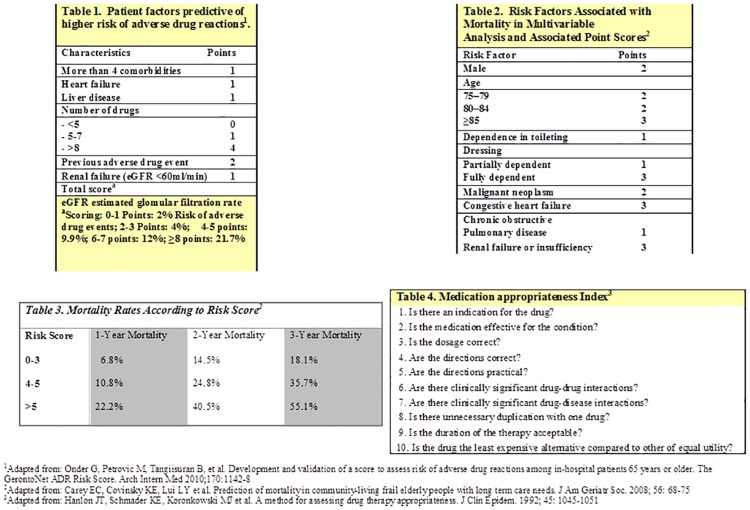
Checklist page 2.

We introduced a checklist (Figs 2 and 3) aimed at supporting the therapeutic reasoning of clinicians in order to improve the quality of drug prescriptions. The checklist was based on the conceptual frameworks proposed by Scott et al. [14] and by Dovjak [15], and consisted of following 5 sequential steps: 1. ascertain all current medications used; 2. identify patients at high risk of adverse drug reactions; 3. estimate life expectancy; 4. identify medications which are not indicated and/or are potentially dangerous; 5. monitor the patient if drugs were stopped or new drugs were added. For the first 4 steps, a possible tool to be used at the discretion of the physician was proposed: for the assessment of current medications: the “brown paper bag” review [16]; to identify patients at high risk of adverse drug reactions: the gerontoNet adverse drug reactions risk score [17]; to estimate life expectancy: the prognostic index for frail elderly people described by Carey et al. [18]; and for the identification of potentially inappropriate medication: the Medication Appropriateness Index [11].

The checklist was discussed and presented to all physicians of the Division of Internal Medicine during grand rounds at the beginning of the intervention period. Each physician received the checklist as a pocket-sized leaflet and was asked to systematically apply the five described steps at admission and discharge of each patient and during daily visits. The checklist was also posted on the mobile desk workstations used during ward rounds. A senior physician reiterated weekly the use of the checklist with the medical team on each ward.

The primary outcomes of the study were the proportion of patients prescribed PIMs at discharge, according to STOPP criteria [19], and the total number of prescribed medications at discharge, before and after the introduction of the checklist. Secondary outcomes (evaluated at discharge), were the prevalence of polypharmacy (concomitant use of ≥ 5 drugs) and hyperpolypharmacy (concomitant use of ≥ 10 drugs), the prevalence of PPOs (according to START criteria [19]), the prevalence of prescription and the rate of inappropriate prescription of following drugs: non-steroidal anti-inflammatory drugs (NSAID); proton pump inhibitors (PPI); systemic corticosteroids; metamizole (Novalgin^™^); potent opiates. In addition we assessed the in-hospital mortality rate and the all-cause re-hospitalization rate at 30 days after discharge.

Basic demographic data and information on diagnoses, medications, duration of hospitalization, admission to the intensive care unit, re-hospitalization within 30 days after discharge, and in-hospital death were collected from the electronic patient records of the hospital. The medication of each patient at admission and discharge was reviewed independently by two of the investigators, who assessed the medication appropriateness by chart review according to STOPP and START criteria [19]. Discrepancies were resolved by discussion. Data were recorded on a standardized case report form and anonymized before statistical analysis. The group assignment of each patient (intervention vs. control) was not reported on the case report form. However, it was not possible to blind the two investigators assessing medication appropriateness, because charts used for chart review contained hospitalization dates, dates of laboratory examinations etc.

Basic demographic characteristics, co-morbidities, clinical and laboratory parameters as well as the type and number of drugs prescribed at admission and discharge were compared according to the intervention using the chi-squared test or Fisher’s exact test for categorical variables as appropriate, and the non-parametric Wilcoxon-Mann-Whitney U test for continuous variables. As the outcome event was common (> 10%) we estimated relative risks and risk ratios (RR) instead of odds ratios (OR), since there might be an overestimation of the effect of the intervention when using OR [20–23]. Because of the failed convergence by the log-binomial logistic method in building multivariate models, we used Poisson regression models with a robust error variance [20] to estimate the effect of the checklist on prescription of at least 20% less drugs at discharge compared to admission, as well as risk factors of prescribing at least one inappropriate drug or missing to prescribe at least one appropriate drug at discharge. Risk factors of being re-hospitalized after discharge were also assessed using Poisson regression models with a robust error variance. Bivariable Poisson regression analysis was used to preselect independent variables when the Wald statistic was p<0.05. If the Phi correlation coefficient between two variables was ≥0.8, the variable with the lowest Wald statistic was excluded from further analysis. Thereafter, we used a backward stepwise multivariable Poisson regression analysis on the selected variables to form the prediction model (entry criteria = p<0.05; removal criteria = p≥0.10). We retained those variables that are known to be associated with the higher probability of drug prescription in the literature (older age, male sex, comorbidities, i.e. Charlton comorbidity index). Likelihood ratio tests were used to measure goodness of the fit of the regression models. Results are presented as crude and adjusted risk ratios (RR) after adjusting for potential confounders as indicated. Finally, we checked the models for any interactions. Data were analyzed using an intention-to-intervention approach, where all subjects in the intervention group were compared regardless of whether checklist has been used by the physician in charge. All analyses were performed using STATA software version 13 for Windows (Stata Corp, College Station, Texas, USA).

The local research ethics committee (Kantonale Ethikkommission Aargau / Solothurn; N. 2013/039) approved the study and accepted the protocol as a quality improvement project aimed at improving the application of recognized standards of care, waiving the requirement to obtain informed consent.

Trial Registration: ClinicalTrials.gov NCT02712268.

## Results

General characteristics of the study population before and after the intervention are shown in Table 1. Overall, in this 900 patients with a median age between 76 (intervention group) and 79 years (control group) 59% of patients were taking > 5 drugs and 13% ≥ 10 drugs at admission. At discharge, after excluding 48 patients who died during hospitalization, the percentages were 72% (patients taking > 5 drugs) and 21% (patients taking ≥ 10 drugs), respectively. About 37% of the 900 patients had ≥ 1 potentially inappropriate medication (PIM) and 25% ≥ 1 missing potentially appropriate medication (PPO) at admission. At discharge, of 852 patients who did not die during the hospitalization, 266 (31%) still had ≥ 1 PIM and 160 (19%) ≥ 1 PPO (Table 1). Overall, at admission 8% of patients had inappropriate prescription of NSAID and 11% inappropriate prescription of PPI (Table 1).

**Table 1:** General characteristics and outcome measures of the 450 patients hospitalized before (“without intervention”) and the 450 patients hospitalized after (“with intervention”) the introduction of the checklist.

Characteristic		Without intervention n = 450		With intervention n = 450		p-value^a^
		n	%	n	%	
Age (median, IQR)		79	73–84	76	71–83	**0.004**
Males		198	44.0	213	47.3	0.31
Living at home		408	90.7	415	92.2	0.40
Number of diagnoses (median, IQR)		8	6–11	7	5–10	**0.001**
Charlson Comorbidity Index	0	86	19.1	94	20.9	0.43
	1–2	197	43.8	195	43.3	
	3–4	115	25.6	98	21.8	
	5	52	11.5	63	14.0	
Number of drugs at admission	<5	172	38.2	196	43.6	0.07
	5–9	208	46.2	205	45.6	
	≥10	70	15.6	49	10.9	
Type of drugs at admission	NSAID	59	13.1	35	7.8	**0.008**
	PPI	149	33.1	130	28.9	0.17
	Corticosteroids	34	7.6	37	8.2	0.72
	Metamizole	13	2.9	9	2.0	0.38
	Opiate	32	7.1	27	6.0	0.50
≥1 potential inappropriate drugs at admission		196	43.7	138	30.6	**<0.001**
≥1 missing appropriate drug at admission		118	26.3	104	23.1	0.26
Inappropriate drugs at admission	NSAID	46	10.2	27	6.0	**0.02**
	PPI	57	12.7	45	9.9	0.20
	Corticosteroids	11	2.5	8	1.8	0.48
	Metamizole	11	2.5	5	1.1	0.10
	Opiate	9	2.0	4	0.9	0.13
Intensive care unit stay		61	13.6	43	9.5	0.06
Hospitalization, days (median, IQR)		7	4–10	6	3–9	**0.02**
Number of drugs at discharge^b^	<5	109	25.9	125	29.0	0.58
	5–9	222	52.7	215	49.9	
	≥10	90	21.4	91	21.1	
Type of drugs at discharge^b^	NSAID	10	2.4	18	4.2	0.14
	PPI	173	41.1	160	37.1	0.24
	Corticosteroids	57	13.5	49	11.4	0.34
	Metamizole	0	-	1	0.2	-
	Opiate	42	10.0	52	12.1	0.33
≥1 potential inappropriate drugs at discharge^b^		164	39.0	102	23.7	**<0.001**
≥1 missing appropriate drug at discharge^b^		88	20.9	72	16.7	0.12
Inappropriate drug at discharge^b^	NSAID	4	1.0	9	2.1	0.14
	PPI	67	15.9	41	9.5	**0.005**
	Corticosteroids	10	2.4	3	0.7	**0.04**
	Metamizole	0	-	1	0.2	-
	Opiate	3	0.7	2	0.5	0.49
Rehospitalization within 30 days^b^		54	12.8	43	10.0	0.19
In-hospital death		29	6.4	19	4.2	0.14

IQR: interquartile range. NSAID: non-steroidal anti-inflammatory drugs. PPI: proton pump inhibitors.

^a^chi-squared test or Fisher’s exact test for categorical variables; Wilcoxon-Mann-Whitney U test for continuos variables.

^b^Total number of patients at discharge = 852 (group without intervention: 421; group with intervention: 431). Total in-hospital death: 48.

Patients in the after-intervention period were younger (median age 76 years vs. 79), had less diagnoses (median number of diagnoses: 7 vs. 8) and a shorter length of stay in the hospital (6 vs. 7 days) than patients in the before-intervention period (Table 1).

The intervention with the checklist was associated with a significant reduction by 22% of the risk of being prescribed ≥ 1 PIM at discharge (adjusted RR 0.78; 95% CI: 0.68–0.94 in the multivariate analysis) (Table 2), but not with a reduction of at least 20% of the number of drugs prescribed at discharge (Table 3), nor with a reduction of the risk of missing potentially appropriate drug prescriptions at discharge (Table 4). A higher risk for prescription of PIM was more likely in patients with PIM at admission, and with an increasing number of diagnoses (test for trend, p<0.001) and medications (test for trend, p<0.001). The analysis of inappropriate prescriptions of NSAID, PPI, corticosteroids, metamizole and opiates at discharge (Table 1) indicated that the intervention with the checklist significantly reduced the risk of inappropriate prescription of PPI at discharge (adjusted RR 0.72, 95% CI: 0.44–0.88, p = 0.010, in the multivariate analysis; data not shown).

**Table 2:** Risk factors for prescription of at least 1 inappropriate drug at discharge.

Variable		Univariate analysis			Multivariate analysis		
		RR	95% CI	p-value^a^	Adj. RR	95% CI	p-value^a^
Age (years)	<70	1	-	-	1	-	-
	70–80	1.12	0.84–1.49	0.439	1.05	0.84–1.31	0.584
	>80	1.27	0.95–1.67	0.106	0.88	0.70–1.10	0.551
Sex	Male	1	-	-	1	-	-
	Female	1.21	0.98–1.48	0.067	1.09	0.93–1.28	0.300
Living condition	At home	1	-	-	1	-	-
	Institutionalized	1.80	1.41–2.31	<0.001	1.21	0.99–1.49	0.068
Charlson Comorbidity Index ≥1		1.08	0.88–1.32	0.480	0.87	0.73–1.03	0.103
Number of diagnoses at admission							
	≤5	1	-	-	1	-	-
	6–8	1.71	1.20–2.43	0.003	0.92	0.68–1.23	0.584
	≥9	2.35	1.69–3.27	<0.001	1.09	0.81–1.48	0.551
Number of drugs at admission							
	≤2	1	-	-	1	-	-
	3–5	2.05	1.24–3.39	0.005	1.24	0.84–1.93	0.334
	6–7	3.91	2.41–6.36	<0.001	1.67	1.09–2.56	**0.018**
	≥8	4.81	3.02–7.68	<0.001	1.68	1.09–2.55	**0.016**
Inappropriate medication at admission		9.86	7.22–13.47	<0.001	8.22	5.96–11.37	**<0.001**
Hospital stay (days), for each additional week		1.13	1.02–1.24	0.018	1.06	0.96–1.15	0.237
Intervention (checklist)		0.61	0.49–0.75	<0.001	0.78	0.68–0.94	**0.005**

RR: risk ratios. CI: confidence interval. Adj. RR: adjusted risk ratios for all variables listed.

^a^Poisson regression models with a robust error variance, Likelihood Ratio Test.

**Table 3:** Predictors for prescription of at least 20% less drugs at discharge compared to admission.

Variable		Univariate analysis			Multivariate analysis		
		RR	95% CI	p-value^a^	Adj. RR	95% CI	p-value^a^
Age (years)	<70	1	-	-	1	-	-
	70–80	1.02	0.61–1.72	0.930	0.99	0.59–1.68	0.991
	>80	1.00	0.59–1.71	0.985	0.83	0.47–1.48	0.537
Sex	Male	1	-	-	1	-	-
	Female	1.07	0.72–1.58	0.731	1.50	0.82–2.72	0.182
Living condition	At home	1	-	-	1	-	-
	Institutionalized	1.70	0.97–2.95	0.062	1.36	1.07–1.74	**0.013**
Charlson Comorbidity Index ≥1		0.89	0.59–1.35	0.593	0.85	0.47–1.31	0.470
Number of diagnoses at admission							
	≤5	1	-	-	1	-	-
	6–8	1.09	0.65–1.85	0.726	0.91	0.52–1.58	0.729
	≥9	1.04	0.62–1.74	0.878	0.85	0.42–1.33	0.333
Number of drugs at admission							
	≤2	1	-	-	1	-	-
	3–5	3.31	1.52–7.23	0.003	3.54	1.62–7.75	**0.002**
	6–7	1.24	0.47–3.25	0.662	1.39	0.52–3.71	0.504
	≥8	3.41	1.55–7.49	0.002	3.94	1.75–8.87	**0.001**
Hospital stay (days), for each additional week		0.99	0.78–1.24	0.924	0.98	0.77–1.25	0.904
Intervention (checklist)		0.80	0.54–1.18	0.266	0.79	0.53–1.16	0.233

RR: risk ratios. CI: confidence interval. Adj. RR: adjusted risk ratios for all variables listed.

^a^Poisson regression models with a robust error variance, Likelihood Ratio Test.

**Table 4:** Risk factors for missing prescription of at least 1 appropriate drug at discharge.

Variable		Univariate analysis			Multivariate analysis		
		RR	95% CI	p-value^a^	Adj. RR	95% CI	p-value^a^
Age (years)	<70	1	-	-	1	-	-
	70–80	1.42	0.93–2.16	0.101	1.32	0.87–1.99	0.187
	>80	1.48	0.97–2.27	0.069	1.23	0.80–1.89	0.351
Sex	Male	1	-	-	1	-	-
	Female	1.11	0.84–1.47	0.467	1.08	0.81–1.45	0.585
Living condition	At home	1	-	-	1	-	-
	Institutionalized	0.99	0.59–1.64	0.959	0.75	0.44–1.26	0.272
Charlson Comorbidity Index ≥1		1.11	0.83–1.48	0.469	0.91	0.67–1.23	0.554
Number of diagnoses at admission							
	≤5	1	-	-	1	-	-
	6–8	1.81	1.10–2.96	0.019	1.53	0.90–2.58	0.113
	≥9	2.61	1.64–4.15	<0.001	1.99	1.19–3.32	**0.008**
Number of drugs at admission							
	≤2	1	-	-	1	-	-
	3–5	1.10	0.65–1.86	0.713	0.94	0.55–1.61	0.821
	6–7	2.13	1.30–3.51	0.003	1.71	1.01–2.89	**0.044**
	≥8	2.26	1.41–3.63	0.001	1.74	1.04–2.91	**0.036**
Hospital stay (days), for each additional week		1.06	0.90–1.23	0.452	0.99	0.83–1.18	0.925
Intervention (checklist)		0.80	0.60–1.06	0.122	0.85	0.65–1.13	0.266

RR: risk ratios. CI: confidence interval. Adj. RR: adjusted risk ratios for all variables listed.

^a^Poisson regression models with a robust error variance, Likelihood Ratio Test.

A higher number of prescribed drugs at admission and not living at home were independently associated with a reduction ≥ 20% of prescribed drugs at discharge (Table 3). A higher number of prescribed drugs and in particular inappropriate medication at admission were associated with a higher risk of prescription of ≥ 1 PIM at discharge (Table 2). A higher number of diagnoses and of prescribed drugs at admission were significantly associated with a higher risk of missing prescription of potentially appropriate medications at discharge (Table 4). The intervention was neither associated with an increased risk of re-hospitalization at 30 days after discharge (Table 5) nor with an increased risk of in-hospital death (Table 6).

**Table 5:** Risk factors for rehospitalization within 30 days after discharge.

Variable		Univariate analysis			Multivariate analysis		
		RR	95% CI	p-value^a^	Adj. RR	95% CI	p-value^a^
Age (years)	<70	1	-	-	1	-	-
	70–80	1.02	0.63–1.67	0.934	0.98	0.60–1.61	0.956
	>80	0.91	0.54–1.52	0.718	0.86	0.50–1.47	0.573
Sex	Male	1	-	-	1	-	-
	Female	1.06	0.72–1.53	0.781	1.17	0.80–1.74	0.416
Living condition	At home	1	-	-	1	-	-
	Institutionalized	0.61	0.26–1.44	0.259	0.61	0.25–1.45	0.264
Charlson Comorbidity Index ≥1		1.58	1.09–2.30	0.016	1.51	0.99–2.30	0.051
Number of diagnoses at admission							
	≤5	1	-	-	1	-	-
	6–8	1.63	0.92–2.89	0.091	1.83	0.99–3.36	0.050
	≥9	1.65	0.94–2.89	0.079	1.68	0.90–3.14	0.101
Number of drugs at admission at admission							
	≤2	1	-	-	1	-	-
	3–5	0.66	0.39–1.11	0.117	0.55	0.32–0.95	**0.031**
	6–7	0.71	0.40–1.28	0.260	0.56	0.31–1.02	0.059
	≥8	0.92	0.56–1.52	0.742	0.68	0.38–1.22	0.202
Hospital stay (days), for each additional week		1.23	1.05–1.44	0.009	1.15	0.97–1.37	0.103
Intervention (checklist)		0.78	0.53–1.13	0.192	0.80	0.55–1.16	0.233

RR: risk ratios. CI: confidence interval. Adj. RR: adjusted risk ratios for all variables listed.

^a^Poisson regression models with a robust error variance, Likelihood Ratio Test.

**Table 6:** Risk factors for in-hospital death.

Variable		Univariate analysis			Multivariate analysis		
		RR	95% CI	p-value^a^	Adj. RR	95% CI	p-value^a^
Age (years)	<70	1	-	-	1	-	-
	70–80	2.12	0.611–7.35	0.236	1.99	0.57–6.96	0.281
	>80	5.69	1.77–18.3	0.004	5.08	1.56–16.5	**0.007**
Sex	Male	1	-	-	1	-	-
	Female	0.99	0.57–1.73	0.981	1.17	0.67–2.04	0.587
Living condition	At home	1	-	-	1	-	-
	Institutionalized	1.82	0.85–3.93	0.124	0.61	0.25–1.45	0.264
Charlson Comorbidity Index ≥1		4.69	2.52–8.75	<0.001	4.94	2.48–9.84	**<0.001**
Number of diagnoses at admission							
	≤5	1	-	-	1	-	-
	6–8	1.71	0.73–3.99	0.216	0.95	0.41–2.35	0.924
	≥9	1.72	0.74–3.97	0.201	0.91	0.37–2.21	0.839
Number of drugs at admission							
	≤2	1	-	-	1	-	-
	3–5	1.60	0.64–3.99	0.308	1.12	0.46–2.68	0.807
	6–7	1.57	0.58–4.23	0.370	0.97	0.37–2.55	0.959
	≥8	1.66	0.66–4.20	0.282	0.73	0.27–1.93	0.530
Hospital stay (days), for each additional week		1.04	0.76–1.42	0.791	0.85	0.57–1.27	0.432
Intervention (checklist)		0.66	0.37–1.15	0.142	0.73	0.43–1.26	0.269

RR: risk ratios. CI: confidence interval. Adj. RR: adjusted risk ratios for all variables listed.

^a^Poisson regression models with a robust error variance, Likelihood Ratio Test.

## Discussion

In our study involving 900 hospitalized patients aged 65 years and older with a high prevalence of polypharmacy, we were able to show that a simple intervention such as the introduction of a 5-steps checklist aimed at supporting the therapeutic reasoning of the treating physician significantly reduced by 22% the risk for the patient of being prescribed ≥ 1 PIM at discharge. Many interventions to improve appropriate prescribing in elderly people have been described in the literature and have been recently reviewed [8, 13, 24]. The effect of our intervention is comparable to the effect of other more complex and time-consuming interventions [8, 13, 24]. Dalleur et al. [25] reported for example that specific STOPP recommendations provided to hospital physicians doubled the reduction of PIMs at discharge in frail inpatients ≥ 75 years old. Recommendations were provided to the ward physician by a geriatrician of an inpatient geriatric consultation team who systematically screened the list of medications on admission for PIMs using STOPP criteria [19]. However, the proportion of patients having ≥ 1 PIM at discharge did not differ between intervention and control group (23% vs. 16%; OR 1.5, 95% CI 0.49–4.89) [25]. In their review Patterson et al. [13] conclude that interventions to improve appropriate polypharmacy, such as pharmaceutical care, appear beneficial in terms of reducing inappropriate prescribing. Eleven of 12 studies included in this review analyzed complex, multi-faceted interventions involving pharmacists and/or specialized physicians (e.g. geriatricians). One intervention consisted of computerized decision support.

Our intervention was not associated with a significant (>20%) reduction of the number of prescribed drugs at discharge (compared to admission) (Adj. RR 0.79, 95% CI 0.53–1.16) (Table 3). This is not surprising, since hospitalized patients are usually treated with additional drugs for the acute problem leading to admission. This result is also in line with former studies showing even an increase in the number of drugs between admission and discharge [26] and points out that polypharmacy is driven by polymorbidity. A higher number of prescribed drugs at admission and not living at home (but in an institution such as e.g. a nursing home) were significantly associated with a reduction ≥ 20% of prescribed drugs at discharge (Table 3). In patients with many medications, the pressure to reduce the number of prescribed drugs may be higher (e.g. because of adverse events, interactions or adherence problems). Moreover, we showed that a higher number of prescribed drugs at admission was significantly associated with a higher risk of prescription of ≥ 1 PIM (Table 2), that should be stopped.

The risk of missing prescription of an appropriate medication at discharge was not reduced by our intervention (Adj. RR 0.85, 95% CI 0.65–1.13) (Table 4). This might be explained by the fact that our intervention primarily focused on reducing inappropriate polypharmacy and the number of prescribed drugs. The introduction of the checklist appeared to be safe, at least in the short term: it was neither associated with an increased risk of in-hospital death nor with an increased risk of re-hospitalization at 30 days after discharge.

We also analyzed the prescription of a few drugs which may be problematic particularly in elderly patients (NSAIDs, systemic corticosteroids, potent opiates), are frequently prescribed often with unclear indication (PPI), or are even considered by several experts to be contraindicated at all, such as metamizole, a controversial NSAID marketed since 1922, that is popular and increasingly used in many countries (including Switzerland), but has been banned in several others (e.g. USA, UK, Canada) because of its association with potentially life threatening agranulocytosis. The introduction of the checklist significantly reduced the risk of inappropriate prescription of PPI at discharge by 28% (adjusted RR 0.72, 95% CI: 0.44–0.88).

We found a high prevalence of polypharmacy (59%) and hyperpolypharmacy (13%), as well as a high rate of potentially inappropriate drug prescriptions (PIM: 37%) and missing potentially appropriate drug prescriptions (PPO: 25%) in 900 patients ≥ 65 years admitted to a division of internal medicine in Switzerland. These results confirm the urgent need of strategies to improve adequacy and safety of drug prescription in elderly patients and are comparable to findings of other studies in other countries. In Europe and Australia the prevalence of polypharmacy and hyperpolypharmacy in elderly hospitalized patients varied in several studies between 52% and 76% for polypharmacy, and between 11% and 24% for hyperpolypharmacy. Between 35% and 77% of patients in these studies were prescribed PIM and the rate of PPO was between 51% and 63% [7, 27–30]. In the USA a cross-sectional study performed in 2007 and including more than 460,000 veterans age 65 and older found that 26% were taking ≥ 1 PIM [3], and among more than 13,000 adults aged ≥ 65 years participating in the National Health & Nutrition Examination Survey the proportion of participants taking ≥ 5 medications tripled from 13% to 39% between 1988 and 2010, while use of PIM decreased from 28% to 15% [1]. Both these US studies included only persons not residing in an inpatient facility and defined PIM according to Beers’ criteria. Only little information was previously available on the situation in Switzerland: among 150 patients aged ≥ 65 years who were admitted to the acute geriatric medicine unit at the Geneva University Hospital 67% were prescribed ≥ 6 and 21% > 10 medications. The PIM prevalence rate was 77% and the PPO rate 65% [27]. A previous study included 800 elderly patients (≥ 65 years) admitted to a general medical or geriatric ward at the University Hospital Basel. The PIM rate according to Beers criteria was overall 18% [31]. Finally, a recent study based on claims data from the largest health insurance in Switzerland and including community-dwelling adults reported for persons older than 65 years a polypharmacy rate (≥ 5 medications) of 41% and a PIM rate of 21% according to 2003 Beers criteria or the PRISCUS list [32]. The lower rates of PIM and polypharmacy reported in the two US studies and in the last two Swiss studies mentioned above in comparison to our results and the cited European and Australian studies may be explained by differences in the examined populations (patients admitted to the hospital versus community-dwelling adults) and by the use of different PIM definitions, since STOPP criteria have a higher sensitivity than e.g. Beers criteria [28, 29].

Our study has several limitations. A randomization was not possible because of the contamination effect, since all physicians of the Division of Internal Medicine rotate on all wards of the division every 1–2 months. In order to compensate for the inherent limitations of a before-after study design we analyzed the most relevant variables influencing drug prescribing, such as indicators of severity of illness and polymorbidity, and accounted for seasonal variations by comparing the patients in the intervention group with patients admitted in the same months of the previous year. Still, the patients in the control group had more diagnoses (8 vs. 7), had a slightly longer hospitalization (median 7 vs. 6 days) and were older (median age 79 vs. 76 years) than in the intervention group. However, we believe that these differences do not reflect relevant differences in the two collectives (e.g. regarding the disease burden), since other more significant characteristics, such as the Charlson Comorbidity Index and the number of drugs at admission were similar. The differences between the two groups might be explained by organizational changes in the Swiss health care system. In 2012 the reimbursement system for the hospitals was switched from a system based on daily fees to the new national Swiss diagnoses related groups (DRG) system, which is a flat rate system providing a fixed remuneration per patient based on diagnoses, procedures, and additional factors (such as age and comorbidities). This system encourages hospitals to shorten length of stay and first analyses suggest that the introduction of the new DRG-system led in Switzerland to a reduction of the duration of hospitalization particularly for elderly patients [33]. In addition, with the new DRG-system also the reimbursement for acute geriatric care and geriatric rehabilitation was changed, leading to the creation in several hospitals (including the Kantonsspital Olten) of new acute geriatric units. It is possible, that during the second part of our study, particularly very old patients have more frequently been admitted to the new acute geriatric unit, than to the division of internal medicine, explaining the difference in age between the study group with and without intervention.

The present study has also several strengths such as the size of the collective studied and the use of STOPP criteria, which require thorough chart-review but appear to be more sensitive and clinically relevant than for example Beers criteria [28, 29].

In conclusion, the introduction of an easy-to-use 5-point checklist aimed at supporting therapeutic reasoning of physicians on internal medicine wards significantly reduced the risk of prescriptions of inappropriate medications at discharge.

## Supporting Information

S1 File Study protocol 19 08 13. (DOC)

S2 File TREND Statement Checklist. (PDF)
