# Supplementary material for: Intervention to Improve Appropriate Prescribing and Reduce Polypharmacy in Elderly Patients Admitted to an Internal Medicine Unit
Source: PLoS One. 2016 Nov 30;11(11):e0166359. doi: 10.1371/journal.pone.0166359 (PMC5130196; doi:10.1371/journal.pone.0166359)
Supplement: S1 File — (DOC) [file pone.0166359.s001.doc]

# Polypharmacy and potentially inappropriate prescribing in elderly adults admitted to an internal medicine unit: is a checklist useful for improving prescribing?

# Urfer Milena^1^, Elzi Luigia^2^, Bassetti Stefano^1^

^1^Department of Internal Medicine, Kantonsspital Olten

^2^Department of Infectious Diseases, University Hospital Basel

# Correspondence:

Prof. Dr. med. Stefano Bassetti

Department of Internal Medicine

Kantonsspital Olten

Baslerstrasse 150

CH – 4600 Olten

Switzerland

e-mail: stefano.bassetti@spital.so.ch

Phone: +41 62 311 42 41

Fax: +41 62 311 41 24

# Introduction

Polypharmacy and the prescribing of inappropriate medication is a common problem in the USA as well as in Europe. Particularly older people are at higher risk for inappropriate medication and consequently for drug related complications (Fick DM, 2008). An increased number of comorbid conditions in the higher aged and therefore a greater number of physicians involved in the care of the elderly put them at higher exposure for polypharmacy and accordingly for inappropriate medication (Gokula M, 2012; Fialová D, 2005). There are at least six major consequences of polypharmacy: nonadherence, adverse drug reactions, drug-drug interactions, increased risk of hospitalisation, medication errors and increased costs (Rollason V, 2003). Studies show a correlation between receiving inappropriate medication and increased morbidity and mortality (Lau D, 2005; Fick DM, 2008). In addition, physiologic changes and impaired organ functions with increasing age lead to altered pharmacokinetics and pharmacodynamics (Beglinger C, 2008; Page RL, 2010). The prescription of the appropriate medication for elderly patients is therefore a challenge for clinicians. The prevalence of polypharmacy and inappropriate medication in Europe is alarming. Fifty-one percent of the elderly in Europe take 6 or more medications and almost 20% of patients use at least one inappropriate medication (Fialová D, 2005). Only few data are available on the situation in Switzerland. A recent prospective European study showed that the prevalence of potentially inappropriate medicines used by patients ≥ 65 years admitted with acute illness to the acute geriatric medicine unit at the Geneva University Hospital was 77.3% (according to STOPP criteria). This was the highest prevalence among the 6 participating European centers (Gallagher P, 2011).

Many interventions aiming at reliably reducing the number of inappropriate medications in elderly patients and at improving the patient’s safety have been studied. Such interventions include for example medication review by multidisciplinary teams or complex multifaceted pharmaceutical care intervention (medication review, consultation, team participation) (Gokula M, 2012; Patterson SM, 2012; Steinman MA, 2010; Topinková E, 2012). Most of these strategies are efficacious for reducing polypharmacy, but are very complex and time-consuming and therefore not easily applicable in the busy clinical daily routine (Gokula M, 2012; Spinewine A, 2007). User-friendly screening tools which can assess the appropriateness of prescribing and do not need a multidisciplinary team do exist. One of the most frequently used is the Beers’ Criteria list, consisting of a list of drugs to avoid in elderly people (Fick DM, 2003). However, most of these tools based on explicit criteria, including the Beers’ Criteria, were developed in the USA or Canada and are hardly applicable in Europe because of the differences in populations, national drug formularies and prescribing attitudes. For example, almost half of the drugs listed in Beers’ criteria are unavailable in European countries (Fialová D, 2005; Gallagher P, 2011). Moreover, explicit (criterion-based) indicators of appropriateness of prescribing are usually drug-oriented or disease-oriented and do not address for example the burden of comorbid disease in the individual patient (Spinewine A, 2007). A research group in Ireland validated a new set of explicit criteria for potentially inappropriate medication use in older adults called STOPP (Screening Tool of Older Person’s Prescriptions) and START (Screening Tool to Alert doctors to Right Treatment) (Gallagher P, 2008). These authors showed also that potentially inappropriate medicines (PIMs) identified according to STOPP criteria and prescribed to patients 65 years or older are significantly associated with avoidable adverse drug events (Hamilton H, 2011). With other tools (implicit approaches) clinicians use information from the patient and published work (instead e.g. of a fixed list of to-avoid-drugs) to make judgements about appropriateness. They address multiple elements of medication prescribing, which are relevant for many different drugs, clinical conditions and settings (Hanlon JT, 1992). These implicit (judgement-based) approaches are flexible, focus on the patient, rather than on drugs or diseases, and are potentially the most sensitive, but they are time-consuming and depend on the user’s knowledge and experience (Hanlon JT, 1992; Spinewine A, 2007).

It is difficult to design an adequate tool which is applicable in the busy clinical routine, respects the complexity and context of the patient and is not limited to a specific region or prescribing habits. We tried to develop such a tool with the aim to improve the appropriateness of prescribing and reduce polypharmacy in an internal medicine unit of a university-affiliated community hospital. We developed a simple checklist which is based on two frameworks proposed in the literature and designed to improve the prescribing process in recognition of patient complexity (Scott IA, 2012; Dovjak P, 2012). These frameworks focus on topics like individual drug-related risk, expected life span, care goals, verification of diagnoses, likely time to benefit, and benefit-risk thresholds of individual drugs and their relative utility (Scott IA, 2012). However, they are complex and require a very detailed and time-consuming evaluation of the patient’s situation and context. We simplified these frameworks and tried to adapt them to the daily clinical routine in order to have a simpler tool aimed at supporting the physician in improving prescribing and reducing polypharmacy.

# Objectives

The primary aim of this study among patients aged ≥ 65 years admitted with an acute illness to an internal medicine unit of a community hospital in north western Switzerland is:

1. To assess the efficacy and safety of a simple prescriber checklist for reducing inappropriate prescribing and polypharmacy.

Secondary aims are:

1. To assess the number of prescribed drugs, the prevalence of polypharmacy (concomitant use of ≥ 5 drugs) and hyperpolypharmacy (concomitant use of ≥ 10 drugs).
2. To assess the prevalence of potentially inappropriate medicine use (PIMs; according to STOPP criteria) and of potentially inappropriate prescribing omissions (PPOs; according to START criteria).
3. To assess the prevalence of prescription and the rate of inappropriate prescription of following drugs: non-steroidal anti-inflammatory drugs (NSAID); proton pump inhibitors (PPI); systemic corticosteroids; metamizol-natrium (Novalgin™); potent opiates.

# Methods

1. ***Study design***

Single-center, interventional, quasi-experimental before-after study.

## Study population

Eligible participants are the first 450 consecutive patients aged ≥ 65 years who will be hospitalised on the internal medicine unit of the Kantonsspital Olten from September 1, 2013 to November 30, 2013. The control group consists of the first 450 consecutive patients aged ≥ 65 years hospitalised on the Internal Medicine Unit of the Kantonsspital Olten between September 1, 2012 and November 30, 2012. As residents and fellows rotate on different wards of the Department of Internal Medicine every 1-2 months, randomization of the intervention to different wards was not possible. Therefore, as ≥ 80% of residents and fellows work in the Department of Internal Medicine for ≥ 2 years, we defined as control group elderly patients hospitalised during the same period one year earlier. We choose the same period to take into account differences in polypharmacy resulting from saisonal variability in prescription of drugs. Each patient will be included only once in the study (at the first hospitalisation during the study period. Patients who are hospitalised during the period 2012 and 2013 will not be considered in the study population 2013).

## Study setting

The Kantonsspital Olten is a university-affiliated community teaching hospital with 245 beds serving ca. 100’000 inhabitants of the eastern part of the Canton of Solothurn, in north western Switzerland. The Department of Internal Medicine comprises an internal medicine unit with 62 beds (ca. 3’300 admissions per year), an acute geriatric unit (45 beds), a palliative care unit (6 beds) and the emergency department. The majority of patients are admitted to the internal medicine unit from the emergency department; the remainder are admitted directly from the community or transferred from other wards or other hospitals.

### *Intervention*

The intervention consists of a checklist (see appendix) given to each physician of the Department of Internal Medicine prior to study start. The checklist will be presented and explained in August 2013 during a “grand round” to all physicians of the department, who will also receive the checklist as a pocket leaflet. The checklist will also be posted on the mobile desk workstations used during ward rounds, and on the intranet. Once a week, a senior physician will reiterate the steps of the prescribing process and the use of the checklist with the medical team on each ward.

### *4.1 Checklist*

The checklist (see appendix) is based on a study by Scott et al. (Scott IA, 2012), who described a 10-step framework to optimize the medication prescription among older people. We used in addition for the configuration of the checklist another eight-step framework proposed under the acronym ACADEMIA (Assess, Comprehensive Geriatric Assessment, Adherence, Development, Emergence, Minimization, Interdisciplinarity, Alertness) (Dovjak P, 2012). We created a simple checklist which should help the physician during busy clinical routine to systematically consider all relevant points for appropriate prescribing, with particular focus on reducing polypharmacy.

Following points are included in the checklist, according to the stepwise process of prescribing (Scott IA, 2012):

1. *Assess current drug use*

The prescriber is encouraged to perform medication reconciliation using the “brown paper bag” method, i.e. asking the patient to bring in all current drugs (Bayoumi I, 2009). This is probably the best way to establish a reliable medication list.

1. *Identify patients at high risk for adverse drug reactions*

The checklist contains a risk score adapted from Carey et al. (Carey EC, 2008). This score indicate the risk of adverse drug reactions according to the presence of 6 patient’s characteristics.

1. *Estimate life expectancy*

The benefits of certain treatments (e.g. treatment with statins for primary prophylaxis of atherosclerosis) may not be realized during the patient’s remaining life span. The estimation of life expectancy is therefore important and has to be taken into account before prescribing a drug. To estimate life expectancy of an individual patient at the bedside is difficult and many factors should be considered. In order to support (but not to replace) this assessment, the checklist contains a risk score adapted from Carey et al. (Carey EC, 2008).

1. *Identification of potentially inappropriate medication*

As a tool for the identification of potentially inappropriate medication, the checklist reports the Medication Appropriateness Index (MAI) (Hanlon JT, 1992). The MAI consists of 10 questions checking indication, dosage, use and costs of a drug.

1. *Monitoring*

The physician is reminded that after changes of therapy or discontinuation of drugs monitoring and re-evaluation are mandatory to rapidly identify possible rebounds of diseases or symptoms, or new medication errors.

*4.2 Use of the checklist*

The medical team on the wards will integrate the use of the described checklist in usual care of all patients hospitalised in the Department of Internal Medicine. The checklist is a tool to improve the application of strategies which are considered standard of care to optimize drug prescriptions and avoid errors or adverse drug events (Scott IA, 2012; Dovjak P, 2012; Meyer-Nikolic VA, 2012).

1. ***Follow-up***

As safety parameter we will assess the all-cause re-hospitalisation rate at 30 days after discharge using administrative data of the hospital. We believe that the re-admission rate calculated only on the basis of re-hospitalisations at the Kantonsspital Olten is representative because the Kantonsspital Olten is the only hospital in the eastern part of the Canton Solothurn and the great majority of patients discharged from the internal medicine unit of the Kantonsspital Olten come back at the same hospital if any new or recurrent problem occur after discharge.

1. ***Data collection***

At the Department of Internal Medicine, Kantonsspital Olten, most patient information (including diagnoses, therapy at admission and discharge, laboratory results, progress notes, etc.) is currently recorded in the electronic patient record. However, some other information, such as one part of the nursing documentation, is still on paper. Data will be collected from the electronic patient records and completed, if necessary, by review of the paper patient records. The medication at admission and discharge of each patient will be reviewed independently by two of the investigators (M.U, S.B), who will assess whether the medication is appropriate or inappropriate according to STOPP and START criteria. Discrepancies in this assessment will be resolved by consensus. Following data will be recorded on a standardised case report form: age; gender; place of residence (home or nursing home); number of diagnoses; Charlson Comorbidity Index (Charlson ME, 1987); length of stay; number of medications at admission and discharge; prescription of NSAID, PPI, systemic corticosteroids, metamizol-natrium, potent opiates, at admission and discharge, and whether these prescriptions are appropriate or not; number of potentially inappropriate medicines (PIM) used and number of potentially inappropriate prescribing omissions (PPO), both at admission and discharge; all-cause re-hospitalisation at 30 days.

Only the investigators will have access to the case report forms, which will be stored in a locked cabinet in the office of the investigators at the Kantonsspital Olten. For statistical analysis data will be anonymised.

1. ***Outcomes***

The primary outcome is the reduction of the proportion of patients using inappropriate drugs (PIM) at discharge and the reduction of the number of prescribed medications at discharge.

Secondary outcomes are:

1. the proportion of patients with potentially inappropriate prescribing omissions (PPOs) at admission and discharge,
2. the prevalence of prescription and the rate of inappropriate prescription of following drugs: non-steroidal anti-inflammatory drugs (NSAID); proton pump inhibitors (PPI); systemic corticosteroids; metamizol-natrium (Novalgin™); potent opiates at admission and discharge,
3. the all-cause rehospitalisation rate at 30 days after discharge

in the intervention and in the control group.

1. ***Sample size***

The sample size is determined by the estimated change in the proportion of users of inappropriate drugs at discharge and the expected size of the intervention effect. Given the high prevalence of polypharmacy in elderly patients (58% in Europe according to Gallagher et al., Gallagher P, 2011) and that 34-77% of these patients use at least one inappropriate drug (PIM), we believe that even a reduction of 10% in patients taking inappropriate medications or a reduction of 10% in the prevalence of polypharmacy will be of clinical relevance. To detect an effect of 10% of the checklist (decrease in prevalence of inappropriate drug prescription and/or polypharmacy from 30% at admission to 20% at discharge) with a probability (power) of 90% at a significance level of 0.05, a sample size of 824 patients (412 in each group) will be needed (table 1.). Therefore, we aim to include 900 patients (450 patients in the intervention group enrolled during the period September-November 2013, and 450 patients in the control group who had been hospitalised in September-November 2012).

1. ***Statistical methods***

Basic demographic characteristics, cumulative co-morbidity (Charlson Co-morbidity Index), the type and number of drugs prescribed and the prevalence of inappropriate prescribing at admission and discharge will be compared using the chi-squared test or Fisher’s exact test for categorical variables, and the Mann-Whitney U test for continuous variables. The results will be analysed with an intention-to-intervention analysis, where all subjects are compared in the intervention group regardless of whether the checklist has been used by the physician in charge. All analyses will be performed using STATA software version 11 for Windows (Stata Corp, College Station, Texas, USA).

1. ***Ethical considerations***

The protocol of the study will be submitted to the local research ethics committee (Kantonale Ethikkommission Aargau / Solothurn). Because the aim of the study is only to evaluate an instrument (the checklist) to improve quality of patient care through better implementation of recognised standards of care (such as medication reconciliation and appropriate prescribing), and data are collected as part of the continuous quality assurance programme of the Kantonsspital Olten, no informed consent from patients will be required.

**Table 1.** Sample size required for each group to detect a 10-20% reduction of the prevalence of inappropriate prescription through a simple checklist according to different power and significance levels.

| Prevalence of polypharmacy or inappropriate drug prescription at admission | Prevalence of polypharmacy or inapproriate drug prescription at discharge | Significance level | Power | Sample size for each group | Total sample size |
| --- | --- | --- | --- | --- | --- |
| 30% | 10% | 0.05 | 90% | 92 | 184 |
| 30% | 20% | 0.05 | 90% | 412 | 824 |
| 20% | 10% | 0.05 | 90% | 286 | 572 |
| 30% | 10% | 0.01 | 90% | 127 | 254 |
| 30% | 20% | 0.01 | 90% | 576 | 1152 |
| 20% | 10% | 0.01 | 90% | 397 | 794 |
| 20% | 10% | 0.01 | 80% | 317 | 634 |

# References

[Bayoumi I](http://www.ncbi.nlm.nih.gov/pubmed?term=Bayoumi I%5BAuthor%5D&cauthor=true&cauthor_uid=19737997), [Howard M](http://www.ncbi.nlm.nih.gov/pubmed?term=Howard M%5BAuthor%5D&cauthor=true&cauthor_uid=19737997), [Holbrook AM](http://www.ncbi.nlm.nih.gov/pubmed?term=Holbrook AM%5BAuthor%5D&cauthor=true&cauthor_uid=19737997), [Schabort I](http://www.ncbi.nlm.nih.gov/pubmed?term=Schabort I%5BAuthor%5D&cauthor=true&cauthor_uid=19737997). Interventions to improve medication reconciliation in primary care. [Ann Pharmacother.](http://www.ncbi.nlm.nih.gov/pubmed?term=Interventions to Improve Medication Reconciliation in Primary Care bay" \l "%23) 2009; 43: 1667-75.

Beglinger, C. Ethics Related to Drug Therapy in the Elderly. Dig Dis. 2008; 26: 28-31.

[Carey EC](http://www.ncbi.nlm.nih.gov/pubmed?term=Carey EC%5BAuthor%5D&cauthor=true&cauthor_uid=18031487), [Covinsky KE](http://www.ncbi.nlm.nih.gov/pubmed?term=Covinsky KE%5BAuthor%5D&cauthor=true&cauthor_uid=18031487), [Lui LY](http://www.ncbi.nlm.nih.gov/pubmed?term=Lui LY%5BAuthor%5D&cauthor=true&cauthor_uid=18031487), [Eng C](http://www.ncbi.nlm.nih.gov/pubmed?term=Eng C%5BAuthor%5D&cauthor=true&cauthor_uid=18031487), [Sands LP](http://www.ncbi.nlm.nih.gov/pubmed?term=Sands LP%5BAuthor%5D&cauthor=true&cauthor_uid=18031487), [Walter LC](http://www.ncbi.nlm.nih.gov/pubmed?term=Walter LC%5BAuthor%5D&cauthor=true&cauthor_uid=18031487). Prediction of mortality in community-living frail elderly people with long-term care needs. J Am Geriatr Soc 2008; 56:68-75.

Charlson ME, Pompei P, Ales KL, MacKenzie CR. A new method of classifying prognostic comorbidity in longitudinal studies: development and validation. Journal of Chronic Diseases 1987; 40: 373-83.

[Dovjak P](http://www.ncbi.nlm.nih.gov/pubmed?term=Dovjak P%5BAuthor%5D&cauthor=true&cauthor_uid=22767400). Tools in polypharmacy. Current evidence from observational and controlled studies. [Z Gerontol Geriatr.](http://www.ncbi.nlm.nih.gov/pubmed/22767400#%23) 2012; 45: 468-72.

[Fialová D](http://www.ncbi.nlm.nih.gov/pubmed?term=Fialová D%5BAuthor%5D&cauthor=true&cauthor_uid=15769968), [Topinková E](http://www.ncbi.nlm.nih.gov/pubmed?term=Topinková E%5BAuthor%5D&cauthor=true&cauthor_uid=15769968), [Gambassi G](http://www.ncbi.nlm.nih.gov/pubmed?term=Gambassi G%5BAuthor%5D&cauthor=true&cauthor_uid=15769968), [Finne-Soveri H](http://www.ncbi.nlm.nih.gov/pubmed?term=Finne-Soveri H%5BAuthor%5D&cauthor=true&cauthor_uid=15769968), [Jónsson PV](http://www.ncbi.nlm.nih.gov/pubmed?term=Jónsson PV%5BAuthor%5D&cauthor=true&cauthor_uid=15769968), [Carpenter I](http://www.ncbi.nlm.nih.gov/pubmed?term=Carpenter I%5BAuthor%5D&cauthor=true&cauthor_uid=15769968). Potentially Inappropriate Medication Use Among Elderly Home Care Patients in Europe. [JAMA](http://www.ncbi.nlm.nih.gov/pubmed/15769968#%23) 2005; 293:1348-58.

[Fick DM](http://www.ncbi.nlm.nih.gov/pubmed?term=Fick DM%5BAuthor%5D&cauthor=true&cauthor_uid=14662625), [Cooper JW](http://www.ncbi.nlm.nih.gov/pubmed?term=Cooper JW%5BAuthor%5D&cauthor=true&cauthor_uid=14662625), [Wade WE](http://www.ncbi.nlm.nih.gov/pubmed?term=Wade WE%5BAuthor%5D&cauthor=true&cauthor_uid=14662625), [Waller JL](http://www.ncbi.nlm.nih.gov/pubmed?term=Waller JL%5BAuthor%5D&cauthor=true&cauthor_uid=14662625), [Maclean JR](http://www.ncbi.nlm.nih.gov/pubmed?term=Maclean JR%5BAuthor%5D&cauthor=true&cauthor_uid=14662625), [Beers MH](http://www.ncbi.nlm.nih.gov/pubmed?term=Beers MH%5BAuthor%5D&cauthor=true&cauthor_uid=14662625). Updating the Beers criteria for potentially inappropriate medication use in older adults: results of a US consensus panel of experts. [Arch Intern Med.](http://www.ncbi.nlm.nih.gov/pubmed?term=Updating the Beers criteria for Potentially Inappropriate Medication Use in Older Adults" \l "%23) 2003; 163: 2716-24.

Fick, DM, Mion, LC; Beers, M. H.; L. Waller, J. Health outcomes associated with potentially inappropriate medication use in older adults. Res. Nurs. Health. 2008; 31: 42-51.

[Gallagher P](http://www.ncbi.nlm.nih.gov/pubmed?term=Gallagher P%5BAuthor%5D&cauthor=true&cauthor_uid=18218287), [Ryan C](http://www.ncbi.nlm.nih.gov/pubmed?term=Ryan C%5BAuthor%5D&cauthor=true&cauthor_uid=18218287), [Byrne S](http://www.ncbi.nlm.nih.gov/pubmed?term=Byrne S%5BAuthor%5D&cauthor=true&cauthor_uid=18218287), [Kennedy J](http://www.ncbi.nlm.nih.gov/pubmed?term=Kennedy J%5BAuthor%5D&cauthor=true&cauthor_uid=18218287), [O'Mahony D](http://www.ncbi.nlm.nih.gov/pubmed?term=O'Mahony D%5BAuthor%5D&cauthor=true&cauthor_uid=18218287). STOPP (Screening Tool of Older Person's Prescriptions) and START (Screening Tool to Alert doctors to Right Treatment). Consensus validation. [Int J Clin Pharmacol Ther.](http://www.ncbi.nlm.nih.gov/pubmed?term=STOPP and START. Consensus validation" \l "%23) 2008; 46: 72-83.

Gallagher P, Lang PO, Cherubini A, Topinková E, Cruz-Jentoft A, Montero Errasquín B. et al. Prevalence of potentially inappropriate prescribing in an acutely ill population of older patients admitted to six European hospitals. Eur J Clin Pharmacol. 2011; 67: 1175–88.

Gokula, M, Holmes, H. M. Tools to Reduce Polypharmacy. Clinics in Geriatric Medicine. 2012; 28: 323-41.

Hamilton H, Gallagher P, Ryan C, Byrne S, O’Mahony D. Potentially inappropriate medications defined by STOPP criteria and the risk of adverse drug events in older hospitalized patients. Arch Intern Med 2011; 171: 1013-19.

[Hanlon JT](http://www.ncbi.nlm.nih.gov/pubmed?term=Hanlon JT%5BAuthor%5D&cauthor=true&cauthor_uid=1474400), [Schmader KE](http://www.ncbi.nlm.nih.gov/pubmed?term=Schmader KE%5BAuthor%5D&cauthor=true&cauthor_uid=1474400), [Samsa GP](http://www.ncbi.nlm.nih.gov/pubmed?term=Samsa GP%5BAuthor%5D&cauthor=true&cauthor_uid=1474400), [Weinberger M](http://www.ncbi.nlm.nih.gov/pubmed?term=Weinberger M%5BAuthor%5D&cauthor=true&cauthor_uid=1474400), [Uttech KM](http://www.ncbi.nlm.nih.gov/pubmed?term=Uttech KM%5BAuthor%5D&cauthor=true&cauthor_uid=1474400), [Lewis IK](http://www.ncbi.nlm.nih.gov/pubmed?term=Lewis IK%5BAuthor%5D&cauthor=true&cauthor_uid=1474400) et al. A method for assessing drug therapy appropriateness. [J Clin Epidemiol.](http://www.ncbi.nlm.nih.gov/pubmed/1474400#%23) 1992; 45:1045-51.

[Lau D. T](http://www.ncbi.nlm.nih.gov/pubmed?term=Lau DT%5BAuthor%5D&cauthor=true&cauthor_uid=15642877), [Kasper J. D](http://www.ncbi.nlm.nih.gov/pubmed?term=Kasper JD%5BAuthor%5D&cauthor=true&cauthor_uid=15642877), [Potter D. E](http://www.ncbi.nlm.nih.gov/pubmed?term=Potter DE%5BAuthor%5D&cauthor=true&cauthor_uid=15642877), [Lyles A](http://www.ncbi.nlm.nih.gov/pubmed?term=Lyles A%5BAuthor%5D&cauthor=true&cauthor_uid=15642877), [Bennett R. G](http://www.ncbi.nlm.nih.gov/pubmed?term=Bennett RG%5BAuthor%5D&cauthor=true&cauthor_uid=15642877). Hospitalization and death associated with potentially inappropriate medication prescriptions among elderly nursing home residents. [Arch Intern Med.](http://www.ncbi.nlm.nih.gov/pubmed/15642877#%23) 2005; 165: 68-74.

Meyer-Nikolic VA, Hersperger M, Herren D. Fehlerquelle Medikamentenverordnung. Schweiz Ärztezeitung 2012; 93: 1595-99.

[Page RL. 2nd](http://www.ncbi.nlm.nih.gov/pubmed?term=Page RL 2nd%5BAuthor%5D&cauthor=true&cauthor_uid=20396637), [Linnebur SA](http://www.ncbi.nlm.nih.gov/pubmed?term=Linnebur SA%5BAuthor%5D&cauthor=true&cauthor_uid=20396637)., [Bryant LL](http://www.ncbi.nlm.nih.gov/pubmed?term=Bryant LL%5BAuthor%5D&cauthor=true&cauthor_uid=20396637)., [Ruscin JM](http://www.ncbi.nlm.nih.gov/pubmed?term=Ruscin JM%5BAuthor%5D&cauthor=true&cauthor_uid=20396637). Inappropriate prescribing in the hospitalized elderly patient: Defining the problem, evaluation tools, and possible solutions. [Clin Interv Aging.](http://www.ncbi.nlm.nih.gov/pubmed?term=Inappropriate prescribing in the hospitalized elderly patient%3A Defining the problem%2C evaluation tools%2C and possible solutions" \l "%23) 2010; 5: 75-87.

[Patterson SM](http://www.ncbi.nlm.nih.gov/pubmed?term=Patterson SM%5BAuthor%5D&cauthor=true&cauthor_uid=22592727), [Hughes C](http://www.ncbi.nlm.nih.gov/pubmed?term=Hughes C%5BAuthor%5D&cauthor=true&cauthor_uid=22592727), [Kerse N](http://www.ncbi.nlm.nih.gov/pubmed?term=Kerse N%5BAuthor%5D&cauthor=true&cauthor_uid=22592727), [Cardwell CR](http://www.ncbi.nlm.nih.gov/pubmed?term=Cardwell CR%5BAuthor%5D&cauthor=true&cauthor_uid=22592727), [Bradley MC](http://www.ncbi.nlm.nih.gov/pubmed?term=Bradley MC%5BAuthor%5D&cauthor=true&cauthor_uid=22592727). Interventions to improve the appropriate use of polypharmacy for older people. [Cochrane Database Syst Rev.](http://www.ncbi.nlm.nih.gov/pubmed/22592727#%23) 2012 May 16;Vol: 5: CD008165.

[Rollason V](http://www.ncbi.nlm.nih.gov/pubmed?term=Rollason V%5BAuthor%5D&cauthor=true&cauthor_uid=12964888), [Vogt N](http://www.ncbi.nlm.nih.gov/pubmed?term=Vogt N%5BAuthor%5D&cauthor=true&cauthor_uid=12964888). Reduction of polypharmacy in the elderly: a systematic review of the role of the pharmacist. [Drugs Aging.](http://www.ncbi.nlm.nih.gov/pubmed?term=reduction of polypharmacy rollason" \l "%23) 2003; 20: 817-32.

[Scott IA](http://www.ncbi.nlm.nih.gov/pubmed?term=Scott IA%5BAuthor%5D&cauthor=true&cauthor_uid=22385783), [Gray LC](http://www.ncbi.nlm.nih.gov/pubmed?term=Gray LC%5BAuthor%5D&cauthor=true&cauthor_uid=22385783), [Martin JH](http://www.ncbi.nlm.nih.gov/pubmed?term=Martin JH%5BAuthor%5D&cauthor=true&cauthor_uid=22385783), [Mitchell CA](http://www.ncbi.nlm.nih.gov/pubmed?term=Mitchell CA%5BAuthor%5D&cauthor=true&cauthor_uid=22385783). Minimizing inappropriate medications in older populations: a 10-step conceptual framework. [Am J Med.](http://www.ncbi.nlm.nih.gov/pubmed?term=Minimizing Inappropriate Medications in Older Populations%3A A 10-step Conceptual Framework" \l "%23) 2012; 125: 529-37.

Spinewine, A, Schmader KE, Barber N, Hughes C, Lapane KL, Swine C, Hanlon JT. Appropriate prescribing in elderly people: how well can it be measured and optimised? Lancet 2007; 370: 173–84.

Steinman MA, Hanlon JT. Managing Medications in Clinically Complex Elders “There's Got to Be a Happy Medium”. JAMA 2010; 304: 1592–601.

[Topinková E](http://www.ncbi.nlm.nih.gov/pubmed?term=Topinková E%5BAuthor%5D&cauthor=true&cauthor_uid=22642782), [Baeyens J.P](http://www.ncbi.nlm.nih.gov/pubmed?term=Baeyens JP%5BAuthor%5D&cauthor=true&cauthor_uid=22642782)., [Michel J.P](http://www.ncbi.nlm.nih.gov/pubmed?term=Michel JP%5BAuthor%5D&cauthor=true&cauthor_uid=22642782)., [Lang P.O](http://www.ncbi.nlm.nih.gov/pubmed?term=Lang PO%5BAuthor%5D&cauthor=true&cauthor_uid=22642782). Evidence-based strategies for the optimiziation of pharmacotherapy in older people. [Drugs Aging.](http://www.ncbi.nlm.nih.gov/pubmed?term=Evidence-Based Strategies for the Optimiziation of Pharmacotherapy in Older People" \l "%23) 2012; 29: 477-94.
